# A Novel Metallo-β-Lactamase Involved in the Ampicillin Resistance of *Streptococcus pneumoniae* ATCC 49136 Strain

**DOI:** 10.1371/journal.pone.0155905

**Published:** 2016-05-23

**Authors:** Chia-Yu Chang, Hui-Jen Lin, Bor-Ran Li, Yaw-Kuen Li

**Affiliations:** 1 Department of Applied Chemistry, National Chiao Tung University, 1001 University Road, Hsinchu, Taiwan; 2 Institute of Biomedical Engineering, National Chiao Tung University, 1001 University Road, Hsinchu, Taiwan; 3 Center for Interdisciplinary Science, National Chiao Tung University, 1001 University Road, Hsinchu, Taiwan; Kaohsiung Medical University, Kaohsiung 80708, Republic of China, TAIWAN

## Abstract

*Streptococcus pneumoniae*, a penicillin-sensitive bacterium, is recognized as a major cause of pneumonia and is treated clinically with penicillin-based antibiotics. The rapid increase in resistance to penicillin and other antibiotics affects 450 million people globally and results in 4 million deaths every year. To unveil the mechanism of resistance of *S*. *pneumoniae* is thus an important issue to treat streptococcal disease that might consequently save millions of lives around the world. In this work, we isolated a *streptococci*-conserved L-ascorbate 6-phosphate lactonase, from *S*. *pneumoniae* ATCC 49136. This protein reveals a metallo-β-lactamase activity *in vitro*, which is able to deactivate an ampicillin-based antibiotic by hydrolyzing the amide bond of the β-lactam ring. The Michaelis parameter (*K*_*m*_) = 25 μM and turnover number (*k*_*cat*_) = 2 s^-1^ were obtained when nitrocefin was utilized as an optically measurable substrate. Through confocal images and western blot analyses with a specific antibody, the indigenous protein was recognized in *S*. *pneumoniae* ATCC 49136. The protein-overexpressed *S*. *pneumonia* exhibits a high ampicillin-tolerance ability *in vivo*. In contrast, the protein-knockout *S*. *pneumonia* reveals the ampicillin-sensitive feature relative to the wild type strain. Based on these results, we propose that this protein is a membrane-associated metallo-β-lactamase (MBL) involved in the antibiotic-resistant property of *S*. *pneumoniae*.

## Introduction

*Streptococcus*, in which cellular division occurs typically along a single axis and which grows in chains or pairs, is among the most common gram-positive bacteria affecting human life. More than 50 *streptococci* species are currently recognized, of which most are facultative anaerobes.[1] *Streptococcus* is in general not a pathogenic microbe; it forms the commensal human microbiome of the mouth, skin, intestine and upper respiratory tract.[2, 3] Particular *streptococcus* species are, however, responsible for many cases of pink eye,[4] meningitis,[5] bacterial pneumonia,[6] endocarditis,[7] erysipelas[8] and necrotizing fasciitis.[9] For example, *S*. *pneumoniae* is recognized to be a major cause of pneumonia.[10]

*S*. *pneumoniae* is typically recognized as the penicillin-sensitive bacterium. Penicillin and other penicillin-like antibiotics (e.g. ampicillin) can efficiently inhibit penicillin-binding proteins (PBP),[11] which are involved in synthesis of the cell wall of a bacterium, leading to irregularities in the *S*. *pneumoniae* cell wall structure such as elongation, lesions, loss of selective permeability and eventual cell death. In addition, penicillin retains comparatively high effectiveness, low cost, ease of delivery and minimal side effects, resulting in a wide usage of penicillin in the therapy of streptococcal disease.[12] The rapid increase in resistance to penicillin[13] and other penicillin-like antibiotics (e.g. ampicillin[14]) is witnessed in both developed and developing countries, which leads to the failure of antibiotic treatment and affects about 450 million people and results in four million deaths every year.[15–17] To discover the drug-resistant mechanism of these pathogenic *streptococci* is thus an important issue for an efficient design of a new drug that might save millions of lives around the world.[18]

The most common mechanism of penicillin resistance generally comes *via* alterations of penicillin-binding proteins (PBP).[19–22] Evolutionary PBP decreasing the penicillin affinity have been recognized as a major resistance mechanism in gram-positive bacteria (e.g. *S*. *pneumoniae*).[23] The other mechanism is to produce β-lactamase that deactivates antibiotics before they effectively approach target sites.[24] Metallo-β-lactamases, capable of hydrolyzing the amide bond of the β-lactam ring in penicillin-based antibiotics, were found to be widely distributed amongst gram-positive and gram-negative bacteria. *S*. *pneumoniae* is thought to elevate the expression of PBP to survive in a penicillin-containing environment.[25–28] In this work, we isolated a *streptococci*-conserved L-ascorbate 6-phosphate lactonase, from *S*. *pneumoniae* ATCC 49136. This protein reveals a metallo-β-lactamase (MBL) activity *in vitro*, which is able to deactivate an ampicillin-based antibiotic by hydrolyzing the amide bond of the β-lactam ring. Our work is designed to shed light on the penicillin resistance and to unveil the possible mechanism and importance of MBL in *streptococci* ecology.

## Materials and Methods

### Chemicals and Materials

LB medium (Tryptone and Yeast extract, Merck KGaA Co., Germany), agar (Oxoid Ltd., UK), ampicillin, penicillin, nitrocefin, kanamycin, isopropyl thiogalactopyranoside (IPTG), and brain-heart infusion (BHI) (Sigma-Aldrich Co., USA), primers (Genomics Co., Taiwan), Pfu DNA Polymerase, dNTP, DNase I and restriction enzymes (New England Biolabs Inc., UK), *S*. *pneumoniae (*Bioresource Collection and Research Center (BCRC) at Food Industry Research and Development Institute (FIRDI, Taiwan) were obtained from the indicated suppliers.

### Gene cloning and sequencing

A gene-encoding *streptococci*-conserved hypothetical protein (locus tag: SMU290, 1089 bp) was isolated from the genome of *S*. *pneumoniae* ATCC 49136 (purchased from Microbiologics® Inc., US) by PCR amplification with primers 5′-TAAGG ATCCG AATTC GATGC CAAAC GTCAA AGAAA TTACA-3′ and 5′-GGATC CTTTT ATAGC AAAGC TTTAA ATTGA AT-3′. The PCR amplification conditions for each cycle were 95°C (0.5 min), 55°C (0.5 min) and 72°C (1.5 min) for 30 cycles in total. The PCR-amplified fragment was then constructed into yT&A pUC18 vector (Yeastern Biotech Co., Taiwan). The plasmid was sequenced with universal primers 5′-GTTTT CCCAG TCACG AC-3′ and 5′-CAGGA AACAG CTATG AC-3′ as sequencing primers. Other plasmids used are stated in a detailed list and summarized in S1 Table and S1 Fig.

### Cell culture

Cells of an *E*. *coli* overexpression system (XL1-blue) were grown in LB medium in an incubator shaker (37°C for 18 h). *Streptococcus* species are grown in a brain-heart infusion in the incubator shaker (37°C, 2 days).

### Protein expression and purification

The cells were harvested using centrifugation at 12,000 x g for 20 min at 4°C. The pellets were stored at -80°C before purification. Cell pellets from 1L LB culture were thawed and resuspended in 10 mL extraction buffer (50 mM Tris-HCl, 200 mM NaCl, and 2 mM β-mercaptoethanol, at pH 8.0) and DNase I (50 units). The cell pellet was disrupted with a cell disrupter (Constant System Ltd, USA). Two cycles of pressure (30 GPa) were applied to destroy the cells. Following removal of the insoluble portion of the crude cell lysate with an ultra-centrifuge (20 min, 75,000 x g, 4°C), the supernatant was passed through a membrane (0.45 μm) and loaded onto an anion- exchange column (Q-column) (GE Healthcare Life Science, USA). The column was washed with 100 mL buffer (25 mM Tris-HCl, at pH 7.0), and eluted with the elution buffer (25 mM Tris-HCl, 500 mM NaCl, at pH 7.0). The active fractions were collected and concentrated with an ultracentrifuge, and further purified with a size-exclusion column (Sephadex G-200, GE Healthcare Life Science, USA). The protein concentration was determined with the Bradford method (Bio-Rad Laboratories, Inc., USA). The aliquot protein was frozen in liquid nitrogen and stored at -80°C.

### Western blot

Western blot analysis of indigenous MBL was performed on loading various whole cell lysates onto a Tris-HCl polyacrylamide gel (15%). Proteins were transferred to a PVDF membrane with Mini Trans-Blot (Bio-Rad Laboratories, Inc., USA) at constant current 400 mA for 2 h. The protein-transferred membranes were blocked with skin milk (5%)/TBST (50 mM Tris 150 mM, NaCl 0.05%, Tween 20, pH 7.6) for 1 h and probed with a rabbit polyclonal antibody (anti-MBL), or anti-6His antibody overnight at 4°C on a rocking platform. Membranes were then washed in TBS-0.1% Tween 20 five times. The horse-Radish peroxidase-conjugated anti-IgG antibody (from rabbit) served as the secondary antibody to probe the primary antibody on the PVDF membrane. Western blotting detection reagent (Amersham ECL Prime) was utilized to develop the signal and monitored with Image Quant LAS 4000 (Amersham, GE Healthcare, USA).

### Immunoprecipitation with nanoparticle

Magnetic Fe_3_O_4_ nanoparticles (MNP) (primary diameter 100 nm, Sigma-Aldrich Co., USA) were dispersed in ethanol solution (50 mL) on ultrasonication for 10 min. 10 mM TEOS dissolved in ethanol solution (50 mL) was then mixed with MNP and stirring at 25°C for 24 h. The reaction products were separated with a centrifuge and washed thoroughly with aqueous ethanol. (3-Aminopropyl)-triethoxysilane solution (1%, APTMS in ethanol) was utilized to functionalize the MNP surface with the amine group at 25°C for 1 h. APTMS-coated MNP were dispersed into 1 mL PBS (phosphate-buffered saline) and then mixed with 50 μL NCS-BA reagent (20 mM isothiocyanato phenylboronic acid in dimethyl sulfoxide, DMSO) at 25°C for 1 h to form a boronic-acid-functionalized MNP (BA@MNP). Through formation of this boronate, the carbohydrate moiety within the constant domain of the SMU290 specific antibody was specifically and covalently linked to BA@MNP.

### Construction of MBL knockout and overexpressed strains

The MBL-mutant of *S*. *pneumoniae* was constructed with insertion mutagenesis with its MBL gene. The suicide vector (pVA891)[29] is the backbone plasmid, which harbors the coding region of the MBL gene inserted at the *Eco*RI restriction site. This constructed plasmid was transformed into the wild type *S*. *pneumoniae*. The MBL gene in pVA891 was inserted into the MBL gene in its genomic DNA with homologous recombination (S2 Fig and S3 Fig). The strains with MBL gene mutation or overexpression were screened from the Erythromycin-containing (10 μg/mL) agar plate after incubation at 37°C for 2 days [30] and further confirmed on re-plating the colonies on a high Erythromycin-containing (100μg/mL) agar plate.

### Enzymatic assays

The enzyme kinetic assays were performed at 25°C in reaction buffer (5 mM Tris-HCl, and 0.1 mM ZnCl_2_, at pH 8.0,) and monitored with a UV-visible spectrophotometer (8453 UV-vis, Agilent Tech., USA). The substrate concentrations were varied between 0.1 and 10 times the *K*_*m*_ value, and the variation of absorbance with time was measured for 60 s at each substrate concentration. *K*_*m*_ and *k*_*cat*_ were determined on fitting data of initial velocity versus substrate concentration directly to the Michaelis equation.

### Analysis of hydrolysis products

The reaction was performed with ampicillin/penicillin (1 mM) and a suitably diluted enzyme (approximately 0.5 μg/mL) at 37°C for 30 min. The supernatant was then analyzed with a mass filter (Q-TOF-1 LC/MS, Micromass, UK) as described in the next section.

### Electrospray ionization-mass spectrometric analysis

Mass spectra were recorded with a quadrupole time-of-flight mass filter (Micromass, UK). This analyzer was scanned over a range 100–2000 u of ratio *m/z* of mass to charge, with scan step 2 s and interscan 0.1 s/step. In these (ESI-MS) experiments, the quadrupole scan mode was conducted under an electrospray voltage 3 kV at the tip of a stainless-steel capillary needle, with source block temperature 80°C. The elution conditions were acetonitrile (50%) containing formic acid (0.1%) at rate 2 μL/min.

### Confocal microscope

Fluorescent images were obtained with a laser-scanning confocal microscope (SP5, Leica., Germany). Green light (488 nm) from a laser was employed to excite the GFP. Throughout the experiments, the power of all lasers, measured before entry into the objective, was kept at _~_ 1 mW. The signal was detected between 495 and 570 nm for GFP. Images were obtained with an objective lens (63X, HCX PLAPO 63XW NA1.20); the image was acquired at 1024 × 1024 pixels (10× optical zoom, 24.6 × 24.6 μm per frame) of scan area with *z*-scan for 0.05 μm during laser irradiation.

## Results and Discussion

### Gene cloning, Protein preparation and Antibody generation

**Fig 1:**
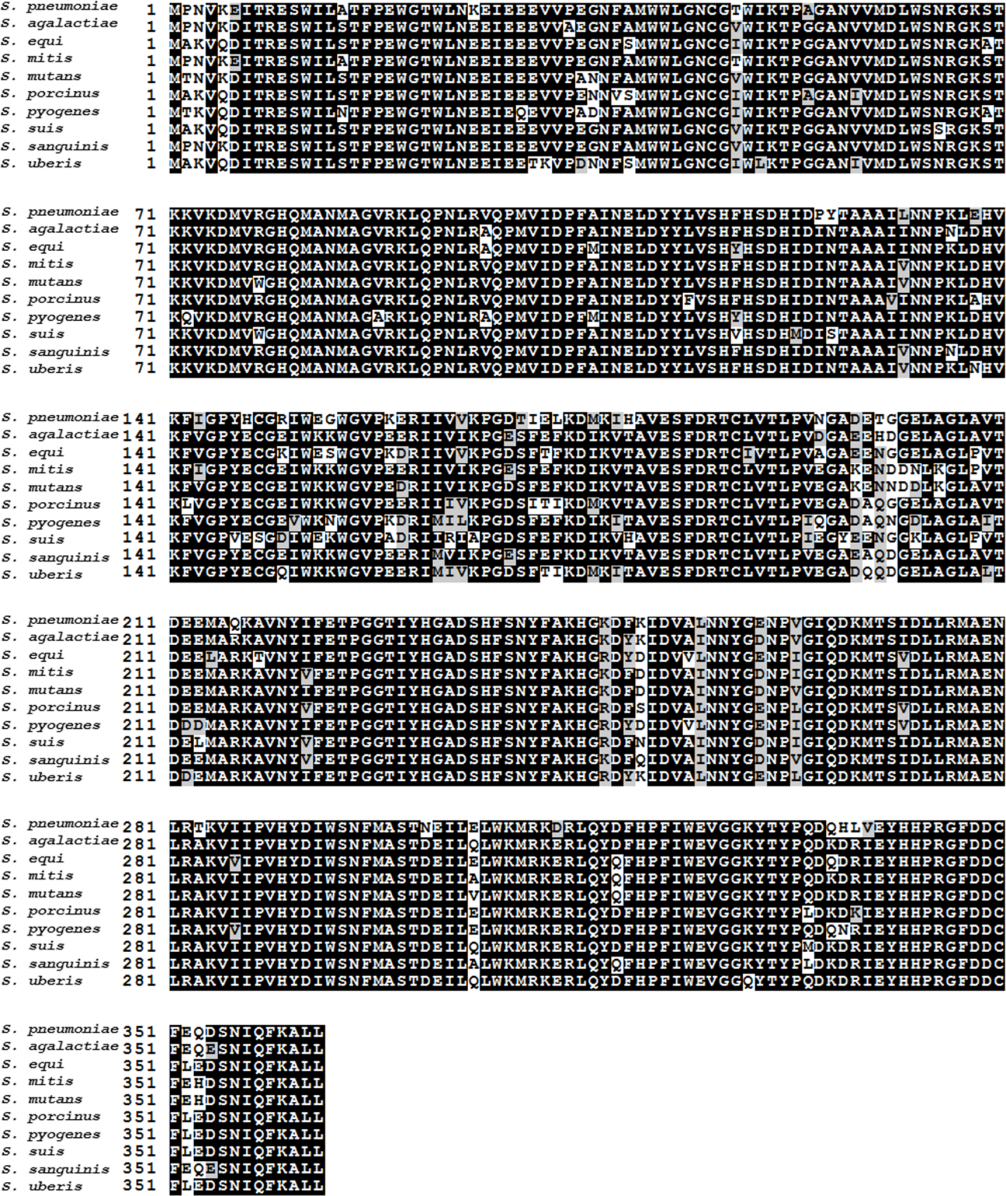
The amino acids sequences alignment of mutliple 6-phosohate ascorbic acid lactonase amino acid from different *Streptococcus* species, including *S*. *pneumoniae* ATCC 49136, *S*. *agalactiae* A909, *S*. *equi* 2329, *S*. *mitis* 21/39, *S*. *mutans* UA159, *S*. *porcinus* Str. Jelinkova 176, *S*. *pyogenes* GA40634, *S*. *suis* R61, *S*. *sanguinis* SK353, and, *S*. *uberis* B362.

As streptococcal metallo-β-lactamase (MBL) is a hypothetical protein with no available experimental information, to prepare MBL protein and MBL-specific antibodies is essential for this work. We amplified the gene encoding this *streptococci*-conserved hypothetical protein from the genome of *S*. *pneumoniae* ATCC 49136 by PCR and subsequently sub-cloned the gene in an expression vector for protein production in *E*. *coli* (XL1-blue). The over-expressed protein, induced by isopropyl thiogalactopyranoside (IPTG), was obtained with high homogeneity (> 95%) (Fig 1) through a series of chromatographic steps (S4 Fig). The molecular mass of the recombinant MBL (363 aa) is consistent with the estimated value ~42 kDa (arrow in S4 Fig). In further experiments this purified protein was employed as the antigen to evoke MBL-specific antibodies from rabbit. This MBL-specific antibody was further utilized in a western blot analysis and fluorescence confocal microscope. Details of the molecular cloning, protein purification and antibody production are described in Materials and Methods.

### Identification of indigenous β-lactamase

Although the gene was predicted to be a metallo-β-lactamase, no information regarding the gene regulation and protein expression in the wild type *streptococci* was discussed. For this reason, the western blotting with a MBL-specific antibody was employed to identify the indigenous MBL in a total cell lysate of *S*. *pneumoniae* ATCC 49136. A band at 42 kDa, corresponding to MBL, could be identified in the cell extract of *S*. *pneumoniae* ATCC 49136. Further imaging examination with a confocal microscope confirms that MBL is expressed and presented on the surface of wild-type *S*. *pneumoniae* ATCC 49136 for which the cell is immunostained with MBL-specific antibody. The results show that the *Streptococci*- conserved MBL, a hypothetical protein, is an activated protein and might play important roles in a cell.

### β-lactamase activity *in vitro*

**Fig 2:**
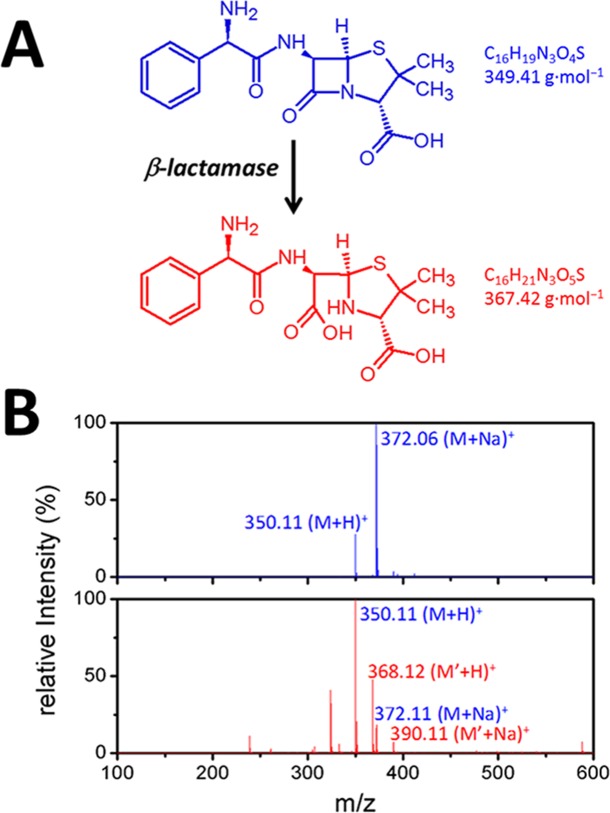
(A) Illustration of the reaction catalyzed by a β-lactamase (B) *m*/*z* values of ampicillin show 350 u (MH)^+^ and 372 u (M-Na^+^) (upper panel). *m*/*z* values of ampicillin after incubating with MBL protein at 25°C for 30 min show new signals at *m*/*z* 368 u (MH)^+^ and 390 u (M-Na^+^) (lower panel).

As the gene of *Streptococci*-conserved MBL is actively expressed, it is of interest to know the biological function of the corresponding protein. From the conserved domain database of NCBI, the gene encoding MBL belongs to the β-lactamase B superfamily (PRK11709),[31] but in the NCBI BLAST database MBL is also predicted to be a L-ascorbate 6-phosphate lactonase.[32] Moreover, the protein structure derived from MBL homologous lactonase reveals an RNase-like metallo-β-lactamase fold.[33] In fact, several lactonases and lactamases are shown to be bifunctional enzymes exhibiting both catalytic activities.[34, 35] MBL, the bioinformatically predicted lactonase, might thus function also as a β-lactamase that can hydrolyze the amide bond of the β-lactam ring (Fig 2A) and allow bacteria to survive in an environment with penicillin-based antibiotics.

To determine β-lactamase activity, ampicillin is utilized as a substrate to confirm the activity of the newly expressed protein.[36] The enzymatic product was analyzed with ESI-mass spectra. As shown in Fig 2B, *m*/*z* of ampicillin is 350 u (MH)^+^ and 372 u (M-Na)^+^; after incubating with MBL protein at 25°C for 30 min, new signals with *m*/*z* 368 u (MH)^+^and 390 u (M-Na)^+^ were observed. The enzymatic product showed a mass shift of +18 Da indicating the hydrolysis of the β-lactam ring of ampicillin. Nitrocefin, a chromogenic cephalosporin substrate routinely used to detect the presence of β-lactamase produced by various microbes,[37] was utilized to confirm the β-lactamase activity of the protein. The hydrolysis of the β-lactam ring of nitrocefin results in a shift of absorption from 395 nm to 498 nm. The maximum absorption coefficient (Δε) of nitrocefin (17936 M^-1^ cm^-1^ at 498 nm) is 20 times that of ampicillin (809 M^-1^ cm^-1^ at 235 nm) or penicillin G (936 M^-1^ cm^-1^ at 235 nm), making it an effective substrate to assay the β-lactamase activity with UV/vis spectra.[38] In S5 Fig, when MBL protein was added to nitrocefin (1 mM), the nitrocefin (395 nm) decreased and hydrolyzed nitrocefin (498 nm) increased with time, indicating that MBL protein is a β-lactamase. The Michaelis parameter (*K*_*m*_) of MBL protein toward nitrocefin is 25 μM; the turnover number (*k*_*cat*_) is 2 s^-1^; the catalytic efficiency (*k*_*cat*_/*K*_*m*_) is 8 × 10^4^ M^-1^ s^-1^, which is compatible with other reported β-lactamase.[38] In addition, no significant β-lactamase activity was observed in the Zn^2+^-free buffer (data not shown), indicating MBL protein is a metallo-β-lactamase.

### Biological function *in vivo*

**Fig 3:**
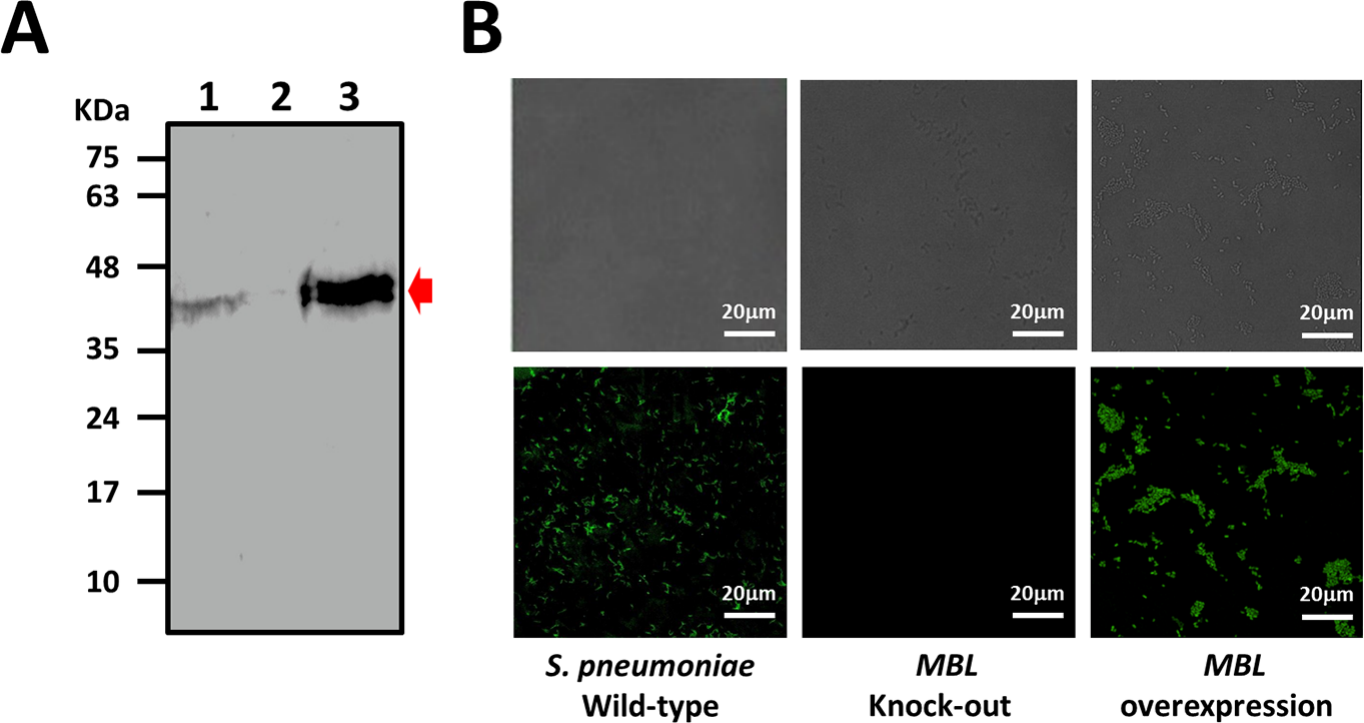
(A) Western blot analysis of MBL protein in the cell extract of the wild type (lane 1), knock-out (lane 2), and MBL-overexpressed (lane 3) *S*. *pneumoniae* strains. A red arrow indicates the MBL bands immunostained with MBL-specific antibody. (B) Brightfield (upper panel) and fluorescence (bottom panel) microscope images demonstrate the presence of MBL in the wild type *S*. *pneumoniae* (ATCC 49136) and MBL overexpression strain, but not in the MBL knock-out strain.

According to the preceding discussion, MBL is confirmed to possess β-lactamase activity. To investigate *in vivo* the biological function of MBL, MBL-knockout and MBL-overexpressed strains were constructed for the phenotype tests. As the result of western blotting shown in Fig 3A, MBL (band at 42 kDa) is identified from the cell extracts of the wild type *S*. *pneumoniae* ATCC 49136 (lane 1) and the MBL-overexpressed strain (lane 3), whereas the MBL is not detectable in the MBL-knockout strain (lane 2). This result is consistent with imaging analysis with a confocal fluorescence microscope (Fig 3B). A green fluorescent image, derived from the binding of FITC-labeled MBL-specific antibody, indicates that MBL is expressed and presented at the cell surface of the wild type and the MBL- overexpressed *S*. *pneumoniae* strains. The absence of fluorescence in the image (at the middle frame) confirms that no detectable MBL is present on the cell surface of the MBL-knockout strain.

**Fig 4:**
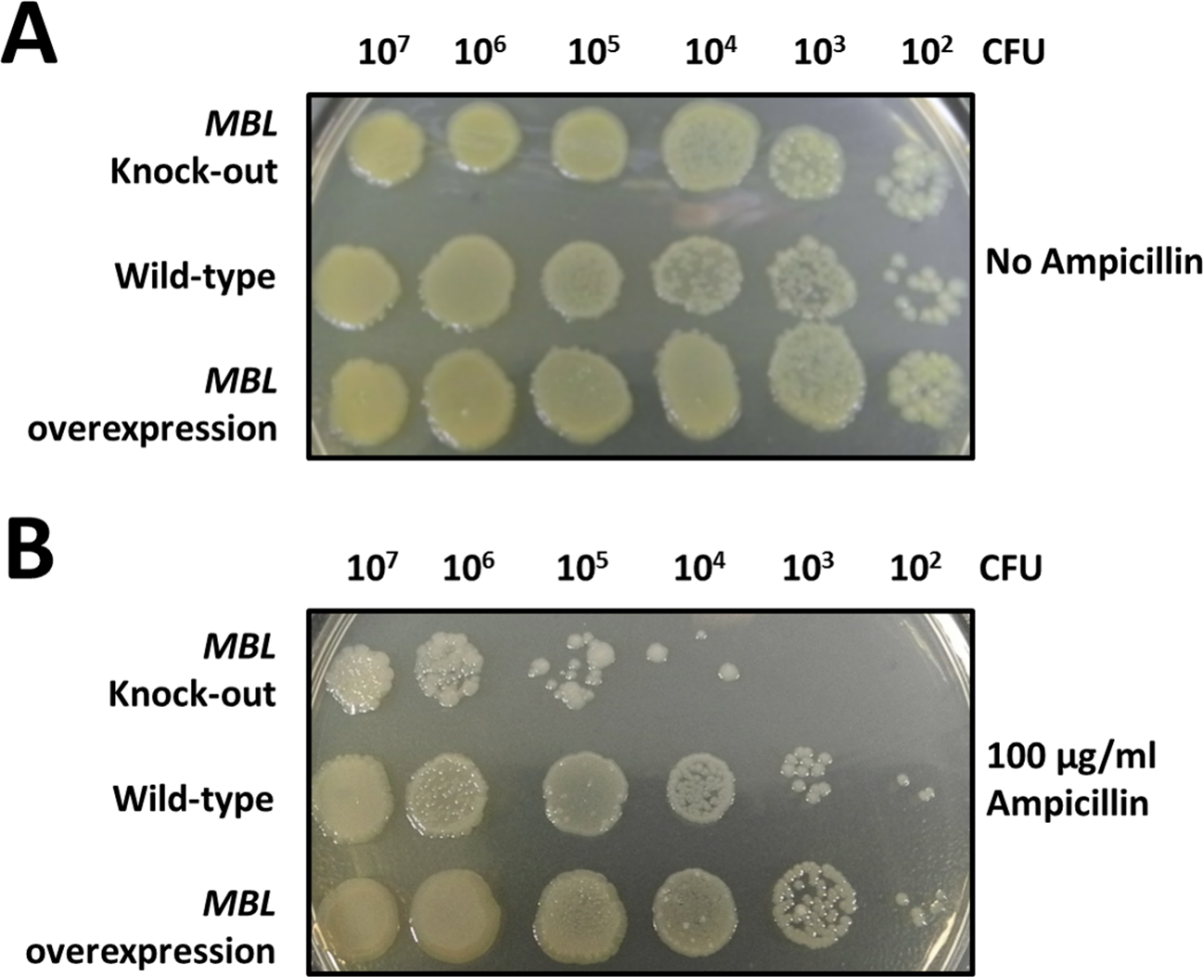
Examination of the ampicillin-resistant ability of wild-type and mutated *S*. *pneumoniae* strains; colonial growth of MBL-knockout, wild-type, and MBL- overexpressed *S*. *pneumoniae* strains (10^2−7^ CFU/mL) on a LB agar plate (A) without, and (B) with ampicillin (100 μM).

We sought further to decide whether the MBL is involved in the ampicillin- resistant property of *S*. *pneumoniae*. A test was conducted to examine the relative rate of survival of the wild type *S*. *pneumoniae*, MBL-knockout, and MBL- overexpressed strains on agar plates with and without ampicillin (100 μM). As the results show in Fig 4A, all strains (10^2−7^ CFU/mL) can grow well on LB agar plates without ampicillin. In contrast, MBL-knockout *S*. *pneumoniae* grows less well than the wild type and MBL-overexpressed strains (Fig 4B). In addition, MBL- overexpressed *S*. *pneumoniae* shows a slightly greater ability of ampicillin tolerance, whereas this condition is lethal for the MBL-knockout strain at a small density of cells (10^2−4^ CFU/mL). All these results indicate strongly that the expression of MBL plays an important role in the ampicillin-resistant *S*. *pneumoniae*.

### MBL in *E*. *coli* system

**Fig 5:**
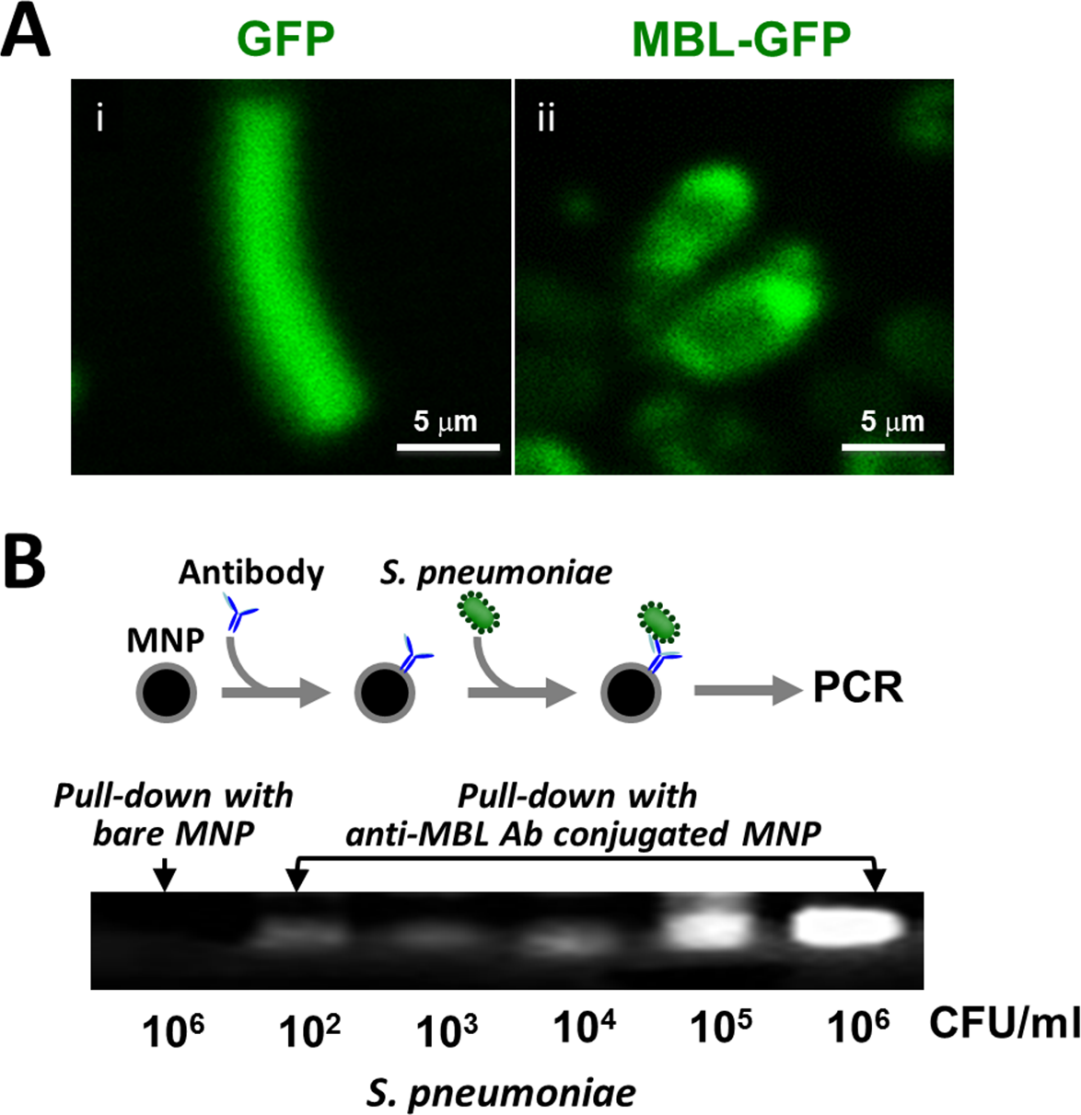
Localization of MBL. (A) Fluorescence microscopic detection of GFP (i) and MBL-GFP (ii) in *E*.*coli*. (B) Upper panel illustrates the immunoprecipitation of bacteria cells with antibody-conjugated magnetic nanoparticles (MNP). Lower panel reveal the PCR products of varied amount of bacteria immunoprecipitated with MBL specific antibody-conjugated magnetic Fe_3_O_4_ nanoparticles (MNP) and subsequent PCR using streptococci-specific primers that recognize the lytA gene.

Subcellular localization is a fundamental and important biological character of a protein, with which an unknown protein can be studied for its targeting or sorting or other biological function. To discover the localization of this novel *streptococci*-conserved metallo-β-lactamase, we transformed plasmids of GFP-fused MBL and GFP-only into the *E*. *coli* system. The expressed GFP-MBL and GFP-only proteins were utilized as indicators to monitor the protein localization with a confocal fluorescence microscope. The results showed that the cellular-expressed GFP-only was homogenously exported in the cytoplasm (Fig 5A), whereas strong fluorescence was observed in surrounding *E*. *coli* cells carrying MBL-GFP plasmid (Fig 5A), indicating that the subcellular localization of MBL is associated with the membrane of a cell. The immunoprecipitate of *S*. *pneumoniae*cell with magnetic iron(III) oxide nanoparticles (MNP) conjugated with MBL specific antibody (Ab-MNP) was utilized to disclose the location (inner or outer membrane) of MBL. If *S*. *pneumoniae* cells were immuno-precipitated with a MBL specific antibody, MBL would be an outer membrane protein. For this reason, *S*. *pneumoniae* cells (10^2−6^ CFU/mL) were separately mixed with Ab-MNP for testing with PCR. According to the result shown in Fig 5B, *S*. *pneumoniae* cells were significantly immuno-precipitated with a MBL-specific antibody. These outcomes strongly indicate MBL to be a protein associated with the outer membrane.

## Conclusions

**Fig 6:**
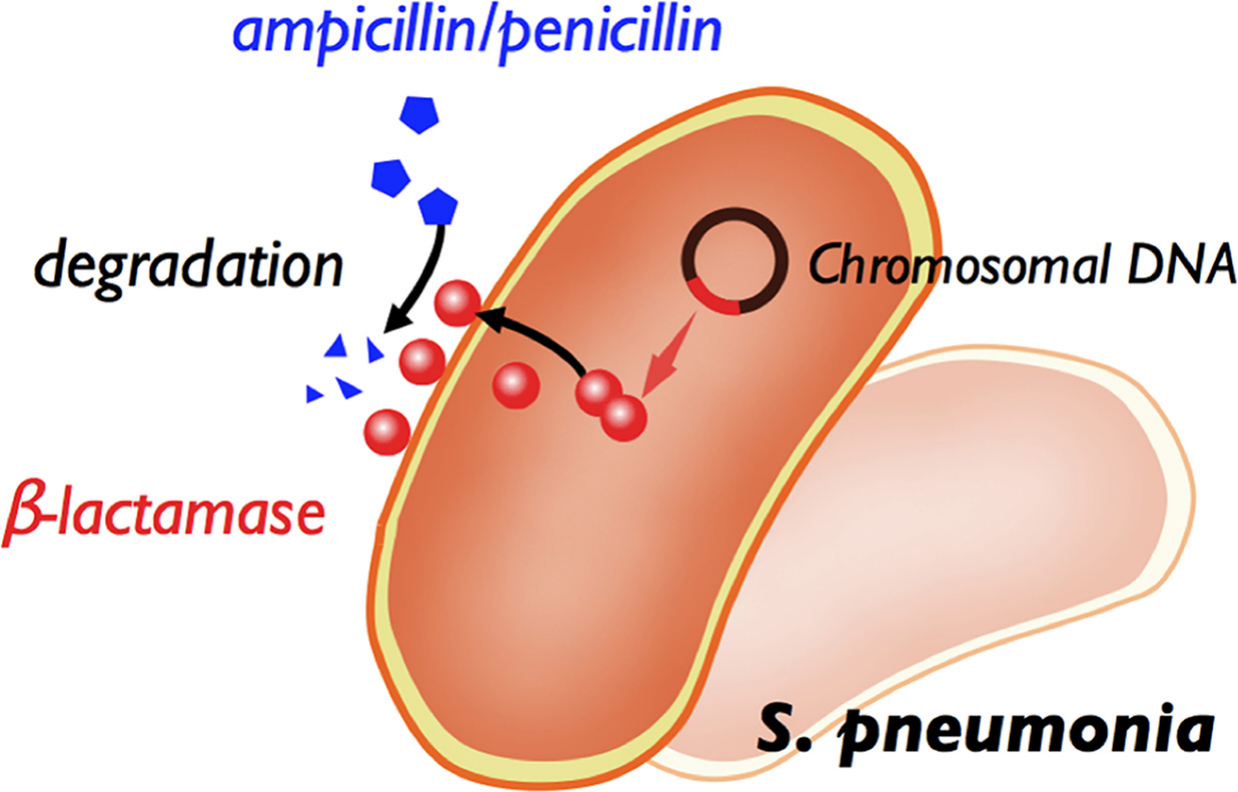
Schemematic illustration of the mechanism of ampicillin resistance of MBL in *S*. *pneumonia* cells.

We report a *streptococci*-conserved gene that encodes a β-lactamase (MBL). According to western blot tests with a MBL-specific antibody, an indigenous MBL is identified in *S*. *pneumoniae* ATCC 49136. Experiments i*n vitro* show that this novel enzyme can deactivate the penicillin-based antibiotics on hydrolyzing the amide bond of the β-lactam ring. The Michaelis parameter (*K*_*m*_) is 25 μM; the catalytic turnover number (*k*_*cat*_) is 2 s^-1^; the catalytic efficiency (*k*_*cat*_/*K*_*m*_) is 8 × 10^4^ M^-1^s^-1^, when nitrocefin is utilized as the substrate. Confocal images of *E*. *coli* bearing the MBL-GFP gene support the conclusion that MBL is a protein associated with the cellular membrane. *In vivo*, the MBL-overexpressed *S*. *pneumonia* strain exhibits a greater ampicillin-tolerant ability. In contrast, the MBL-knockout *S*. *pneumonia* strain reveals the ampicillin-sensitive properties relative to the wild type strain. All these results strongly indicate that the expression of MBL is important for *S*. *pneumoniae* to survive in an ampicillin-containing environment. Based on these findings, we propose that the metallo-β-lactamase (MBL) is, at least partially, involved in the penicillin-resistant character of *S*. *pneumoniae* (PRSP). The mechanism of antibiotic resistance of *S*. *pneumoniae* might result from, at least in part, the enzymatic degradation of penicillin-based drugs on the surface of the bacterial cell as depicted in Fig 6. This striking finding can be possibly employed to develop a specific diagnosis and targeted drug delivery towards *S*. *pneumoniae*.

## Supporting Information

S1 Fig Illustration of the maps of plasmids used in this work (PDF)

S2 Fig Illustration of the construction of MBL overexpressed *S. pneumoniae* strain (PDF)

S3 Fig Illustration of the construction of MBL knockout *S. pneumoniae* strain (PDF)

S4 Fig SDS-PAGE analysis of fractions from various steps of MBL purification. (PDF)

S5 Fig UV-vis differential absorption of nitrocefin hydrolyzed with SMU290 protein. (PDF)

S6 Fig Kinetic analysis of nitrocefin hydrolyzed by metallo-β-lactamase. (PDF)

S1 Table List of Plasmids (PDF)
