# Supplementary figures and images for: A Novel Metallo-β-Lactamase Involved in the Ampicillin Resistance of Streptococcus pneumoniae ATCC 49136 Strain
Source: PLoS One. 2016 May 23;11(5):e0155905. doi: 10.1371/journal.pone.0155905 (PMC4877090; doi:10.1371/journal.pone.0155905)

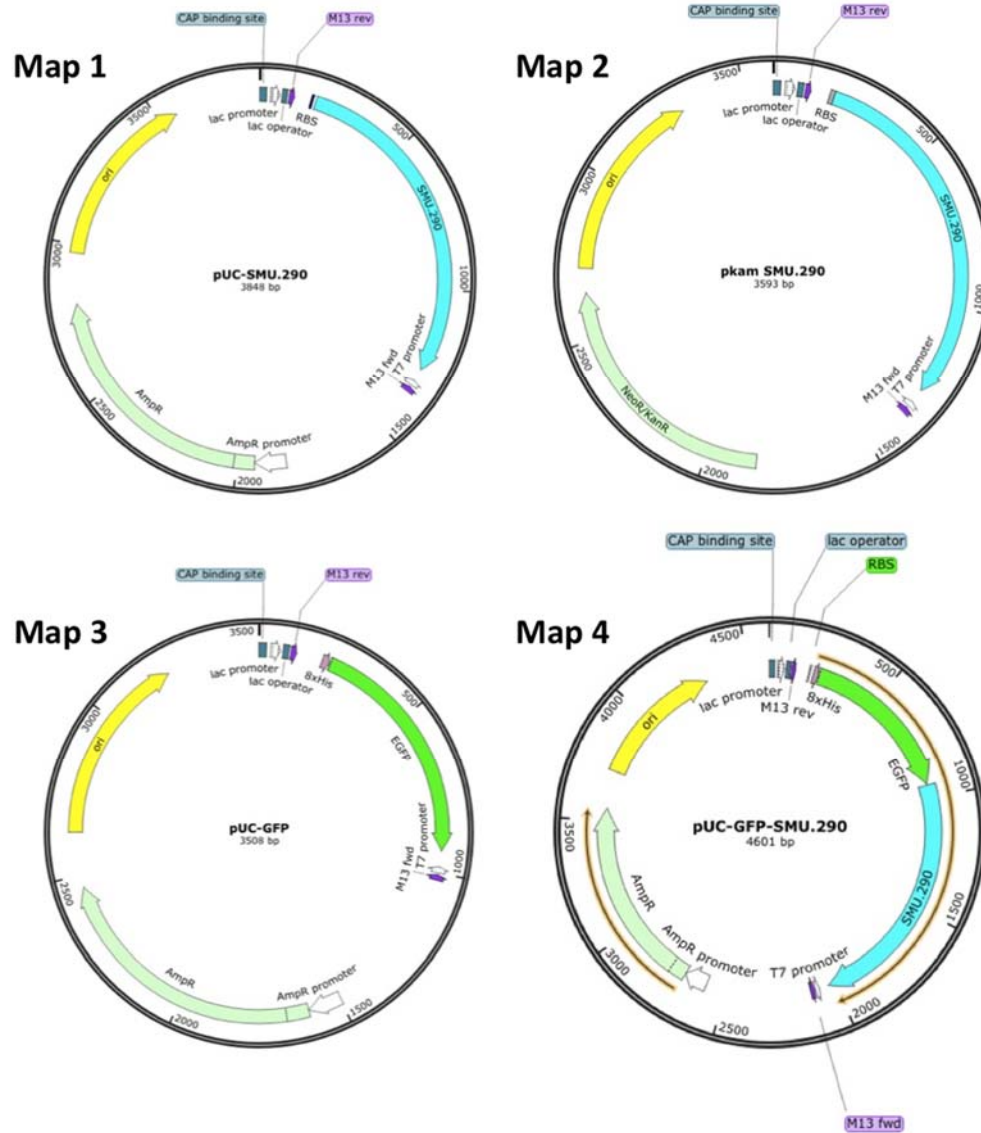

S1 Fig. Illustration of the maps of plasmids used in this work

Supplement: S1 Fig — (PDF) [file pone.0155905.s001.pdf]
