# Supplementary material for: A Novel Metallo-β-Lactamase Involved in the Ampicillin Resistance of Streptococcus pneumoniae ATCC 49136 Strain
Source: PLoS One. 2016 May 23;11(5):e0155905. doi: 10.1371/journal.pone.0155905 (PMC4877090; doi:10.1371/journal.pone.0155905)
Supplement: S2 Fig — (PDF) [file pone.0155905.s002.pdf]

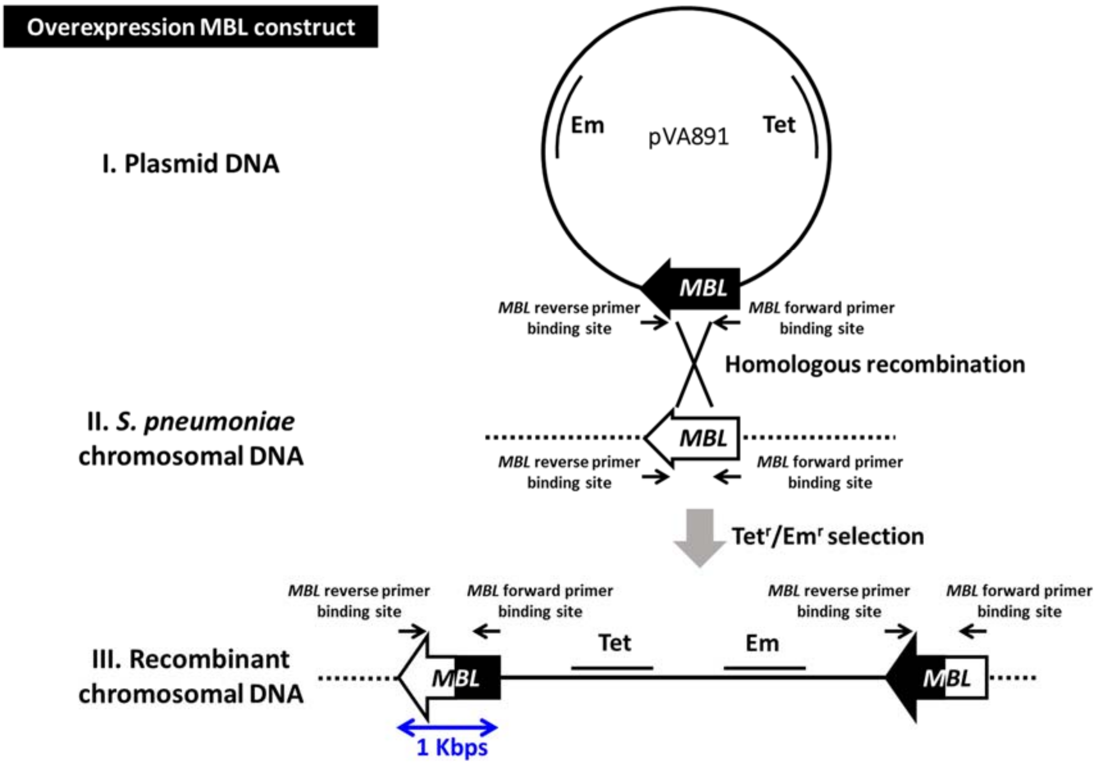

S2 Fig. Illustration of the construction of MBL overexpressed *S. pneumoniae* strain
