# Supplementary material for: A Novel Metallo-β-Lactamase Involved in the Ampicillin Resistance of Streptococcus pneumoniae ATCC 49136 Strain
Source: PLoS One. 2016 May 23;11(5):e0155905. doi: 10.1371/journal.pone.0155905 (PMC4877090; doi:10.1371/journal.pone.0155905)
Supplement: S4 Fig — (PDF) [file pone.0155905.s004.pdf]

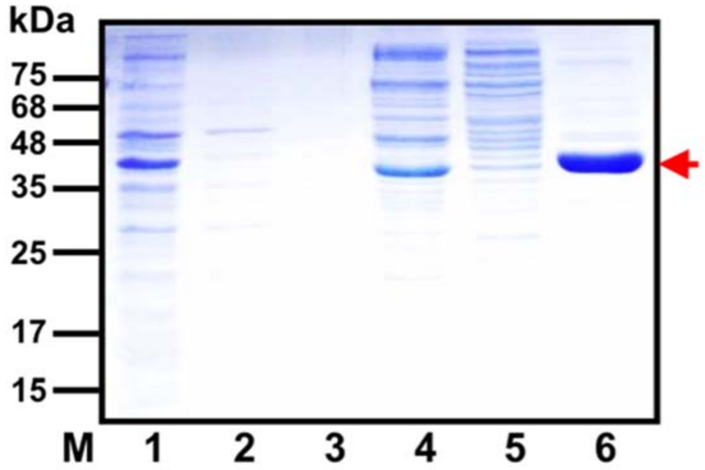

S4 Fig. SDS-PAGE analysis of fractions from various steps of MBL purification. Lane 1: soluble fraction after cell disruption; lane 2: unbound fraction passed through Q-column; lane 3: fraction washed from column with PBS; lanes 4-6: fractions of elution with phosphate buffer (20 mM, pH 7.2) containing NaCl (100, 200 and 500 mM), individually. A red arrow indicates the band of the MBL protein.
