# Supplementary material for: A Novel Metallo-β-Lactamase Involved in the Ampicillin Resistance of Streptococcus pneumoniae ATCC 49136 Strain
Source: PLoS One. 2016 May 23;11(5):e0155905. doi: 10.1371/journal.pone.0155905 (PMC4877090; doi:10.1371/journal.pone.0155905)
Supplement: S5 Fig — (PDF) [file pone.0155905.s005.pdf]

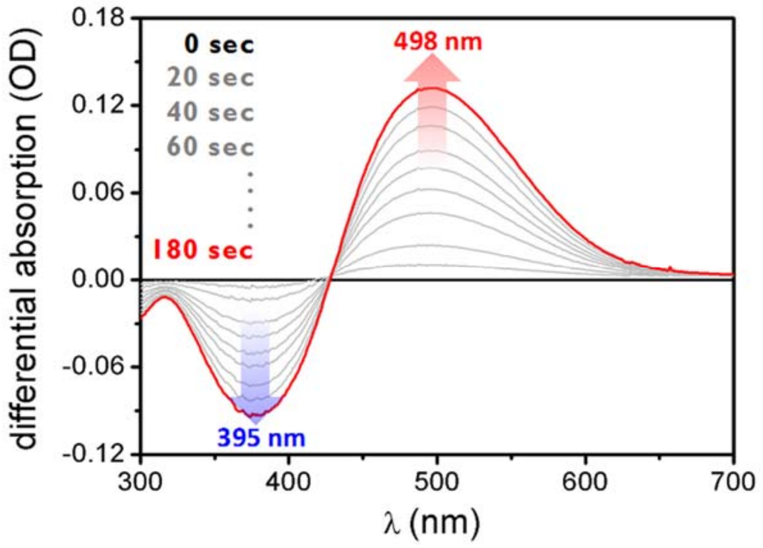

S5 Fig. Temporal course (0 to 180 s) UV-vis differential absorption of nitrocefin hydrolyzed with SMU290 protein. The nitrocefin signal (395 nm) decreased and the signal of hydrolyzed nitrocefin (498 nm) increased with time.
