# Supplementary material for: A Novel Metallo-β-Lactamase Involved in the Ampicillin Resistance of Streptococcus pneumoniae ATCC 49136 Strain
Source: PLoS One. 2016 May 23;11(5):e0155905. doi: 10.1371/journal.pone.0155905 (PMC4877090; doi:10.1371/journal.pone.0155905)
Supplement: S6 Fig — (PDF) [file pone.0155905.s006.pdf]

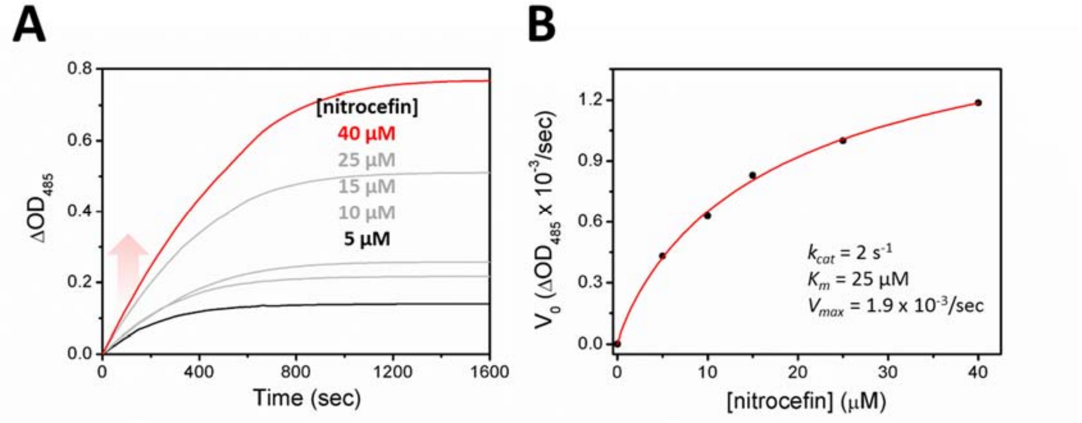

S6 Fig. Steady-state Michaelis–Menten analysis of nitrocefin hydrolyzed by metallo-β-lactamase. (A) Typical kinetic experiments performed at 25.0°C in PBS, pH 7.0, containing 5mM $ZnCl_2$, and UV spectrum is utilized to record hydrolyzed nitrocefin at 485 nm (absorption coefficient (ε) = 20,500 M^{-1} cm^{-1}). (B) The initial velocities are recorded as a function of nitrocefin concentration. The data are following fit to Michaelis equation to derive the kinetic parameters k_{cat}, K_m, and V_{max} through OriginPro 8 software.
