# Supplementary material for: A Novel Metallo-β-Lactamase Involved in the Ampicillin Resistance of Streptococcus pneumoniae ATCC 49136 Strain
Source: PLoS One. 2016 May 23;11(5):e0155905. doi: 10.1371/journal.pone.0155905 (PMC4877090; doi:10.1371/journal.pone.0155905)
Supplement: S1 Table — (PDF) [file pone.0155905.s007.pdf]

S1 Table. List of Plasmids

Plasmid name	Selection marker	Replication origin	Experiment	Maps
pUC-MBL	Ampicillin	p15A	Protein over expression	1
pkam-MBL	Kanamycin	p15A	Antibiotic resistant test	2
pUC-GFP	Ampicillin	p15A	Protein localization	3
pUC-GFP-MBL	Ampicillin	p15A	Protein localization	4
